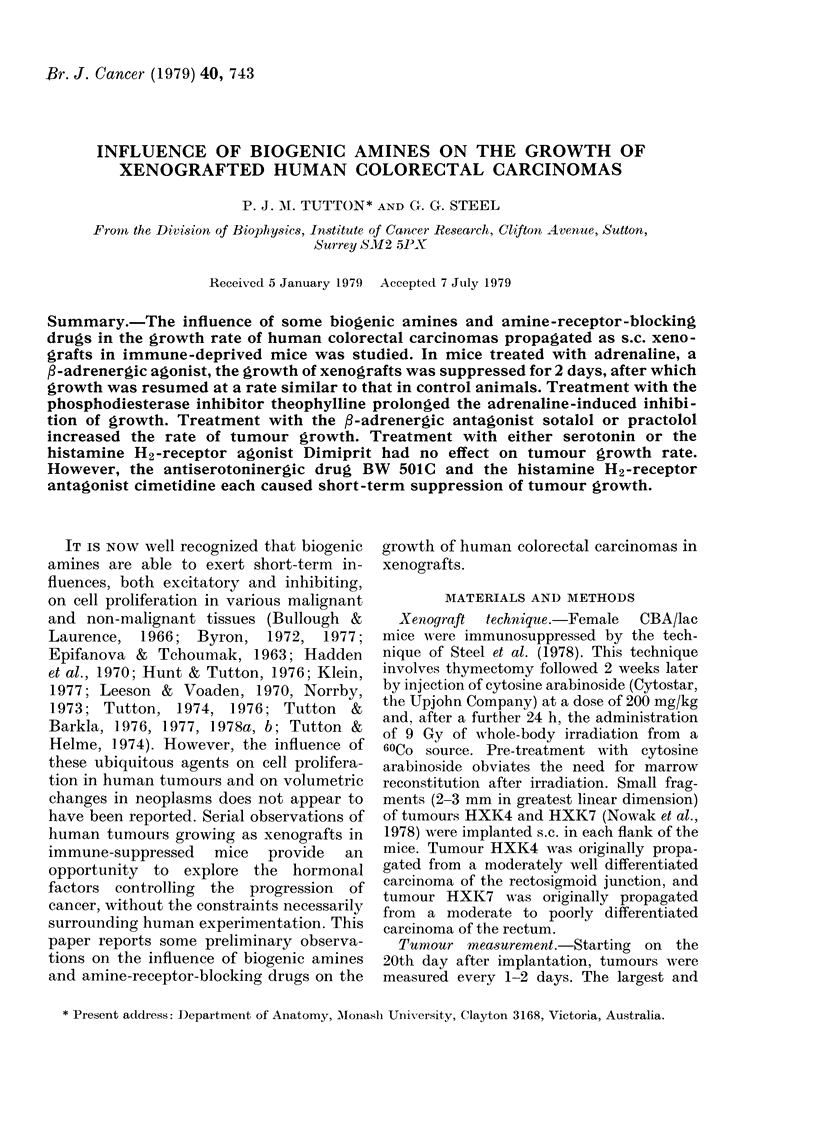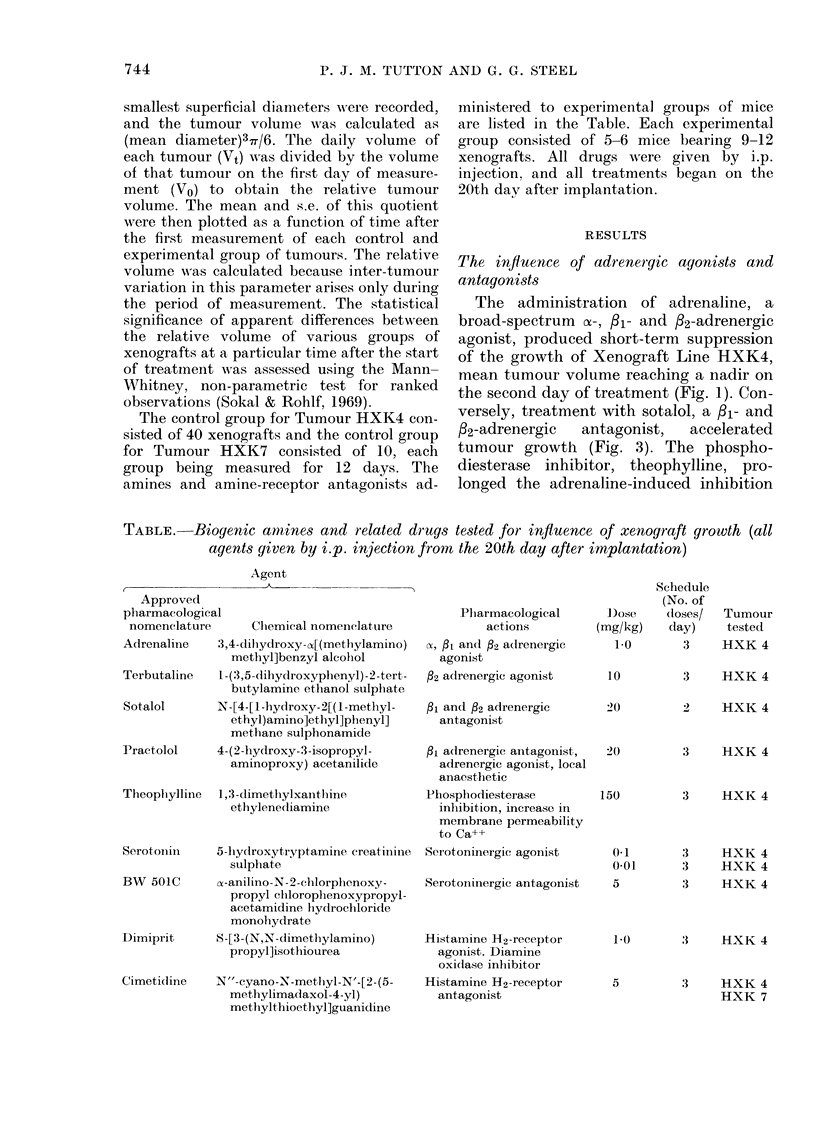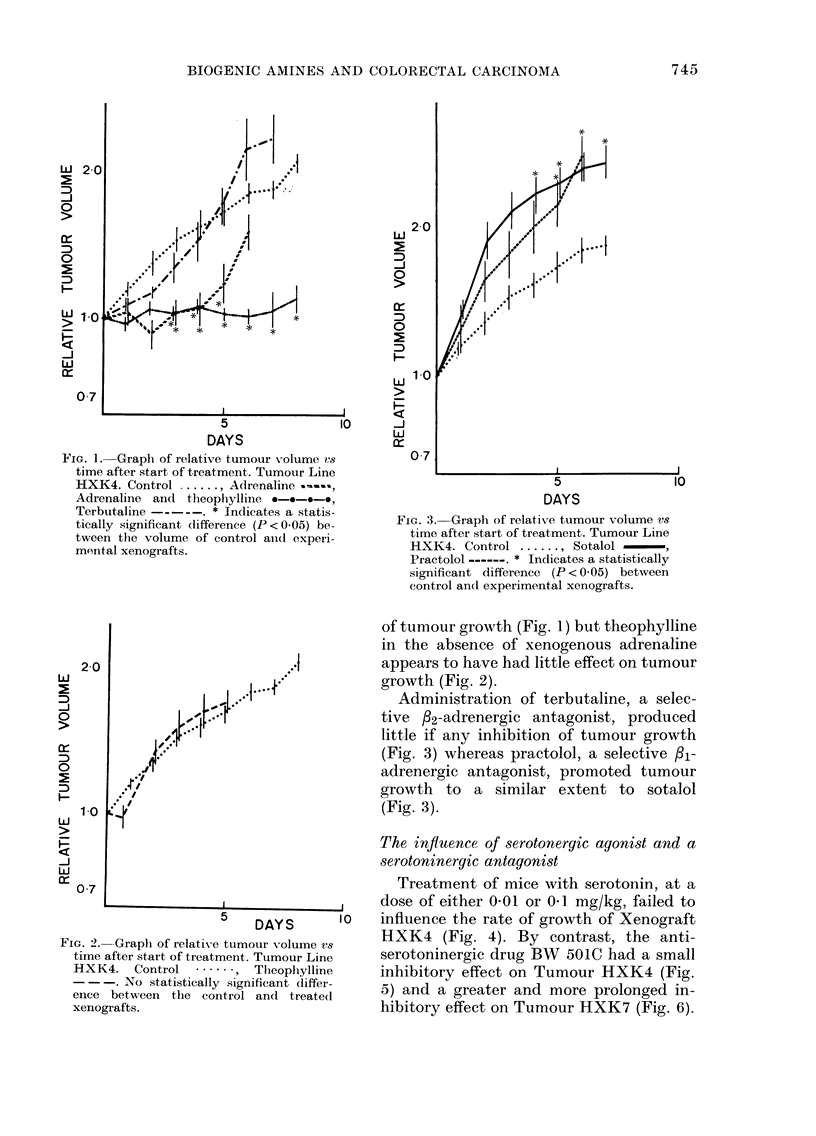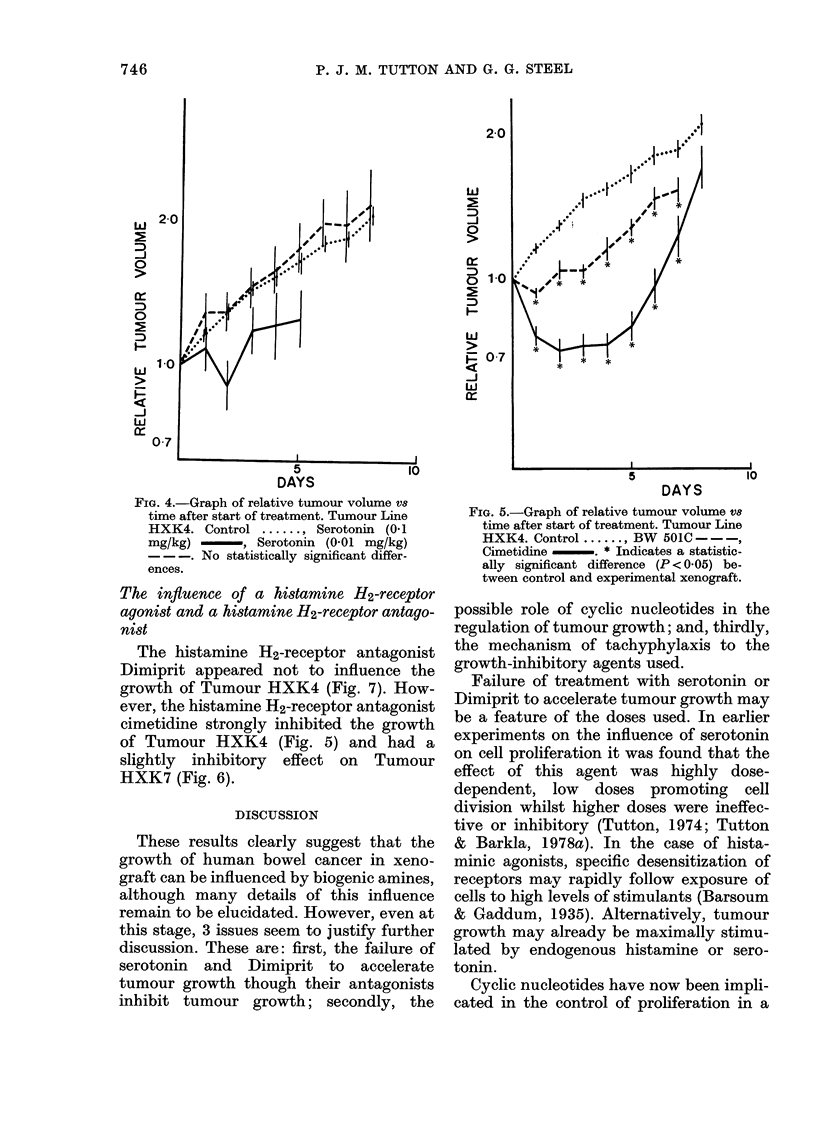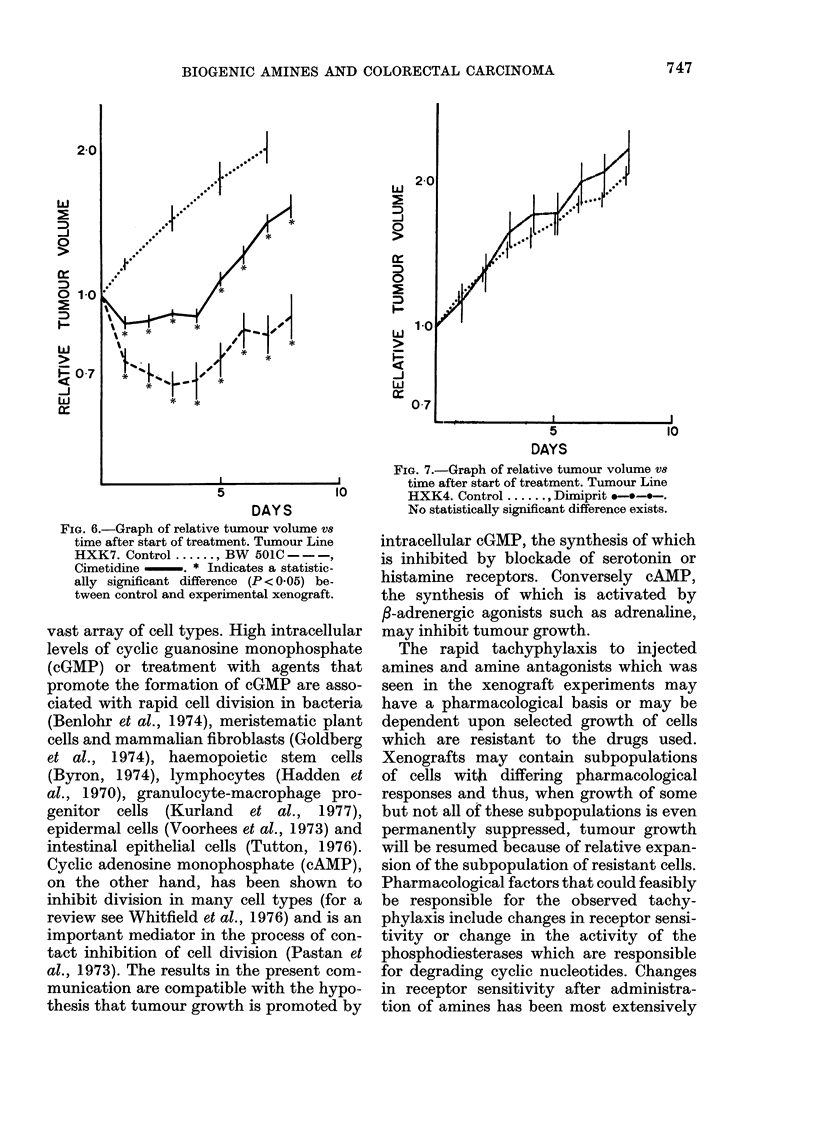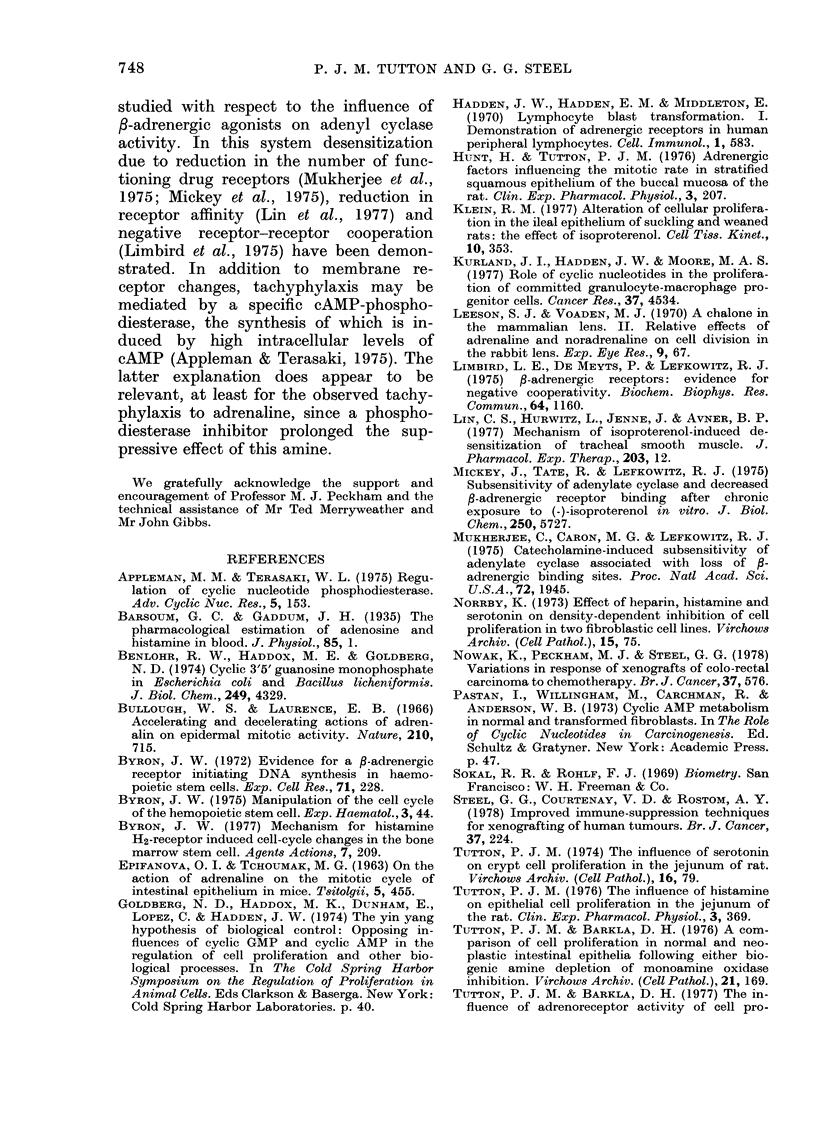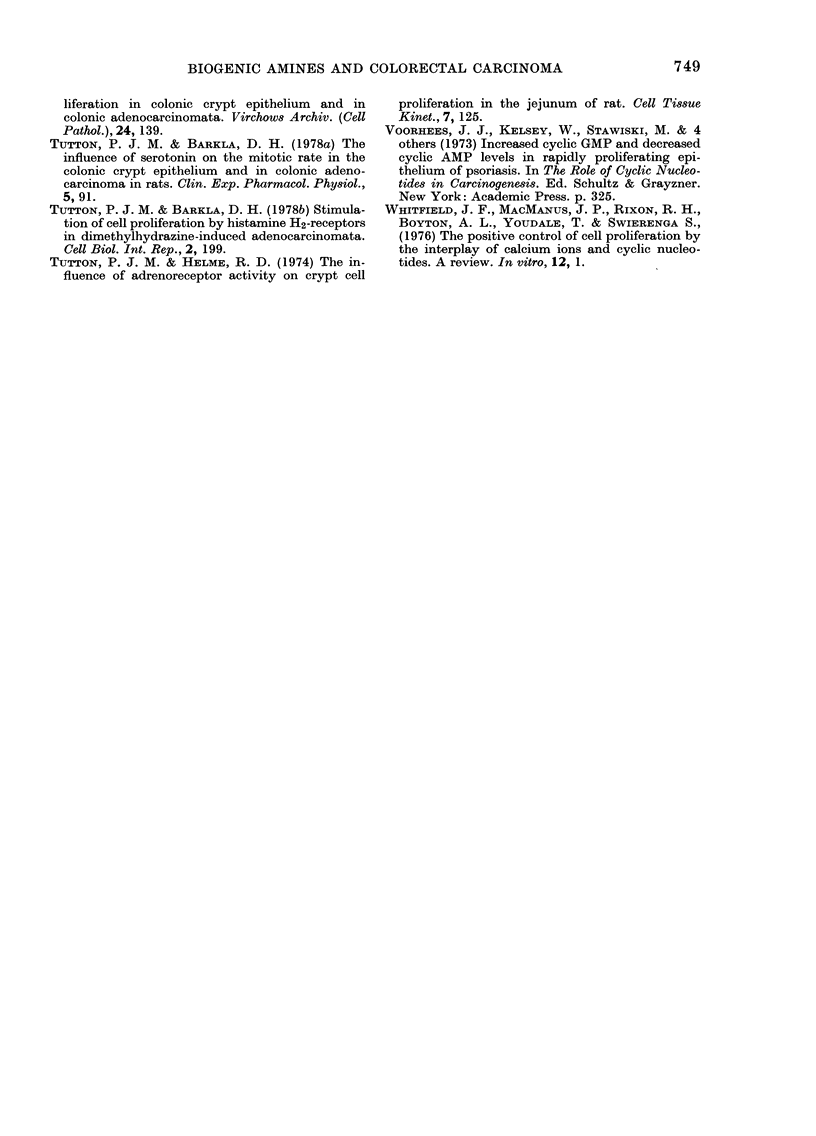# Influence of biogenic amines on the growth of xenografted human colorectal carcinomas.

**DOI:** 10.1038/bjc.1979.255

**Published:** 1979-11

**Authors:** P. J. Tutton, G. G. Steel

## Abstract

The influence of some biogenic amines and amine-receptor-blocking drugs in the growth rate of human colorectal carcinomas propagated as s.c. xenografts in immune-deprived mice was studied. In mice treated with adrenaline, a beta-adrenergic agonist, the growth of xenografts was suppressed for 2 days, after which growth was resumed at a rate similar to that in control animals. Treatment with the phosphodiesterase inhibitor theophylline prolonged the adrenaline-induced inhibition of growth. Treatment with the beta-adrenergic antagonist sotalol or practolol increased the rate of tumour growth. Treatment with either serotonin or the histamine H2-receptor agonist Dimiprit had no effect on tumour growth rate. However, the antiserotoninergic drug BW 501C and the histamine H2-receptor antagonist cimetidine each caused short-term suppression of tumour growth.


*Br. J. Cancer* (1979) **40**, 743

### INFLUENCE OF BIOGENIC AMINES ON THE GROWTH OF XENOGRAFTED HUMAN COLORECTAL CARCINOMAS

P. J. M. TUTTON* AND G. G. STEEL

*From the Division of Biophysics, Institute of Cancer Research, Clifton Avenue, Sutton, Surrey SM2 5PX*

Received 5 January 1979 Accepted 7 July 1979

**Summary.—The influence of some biogenic amines and amine-receptor-blocking drugs in the growth rate of human colorectal carcinomas propagated as s.c. xenografts in immune-deprived mice was studied. In mice treated with adrenaline, a $\beta$-adrenergic agonist, the growth of xenografts was suppressed for 2 days, after which growth was resumed at a rate similar to that in control animals. Treatment with the phosphodiesterase inhibitor theophylline prolonged the adrenaline-induced inhibition of growth. Treatment with the $\beta$-adrenergic antagonist sotalol or practolol increased the rate of tumour growth. Treatment with either serotonin or the histamine $H_2$-receptor agonist Dimiprit had no effect on tumour growth rate. However, the antiserotoninergic drug BW 501C and the histamine $H_2$-receptor antagonist cimetidine each caused short-term suppression of tumour growth.**

It is now well recognized that biogenic amines are able to exert short-term influences, both excitatory and inhibiting, on cell proliferation in various malignant and non-malignant tissues (Bullough & Laurence, 1966; Byron, 1972, 1977; Epifanova & Tchoumak, 1963; Hadden *et al.*, 1970; Hunt & Tutton, 1976; Klein, 1977; Leeson & Voaden, 1970, Norrby, 1973; Tutton, 1974, 1976; Tutton & Barkla, 1976, 1977, 1978*a*, *b*; Tutton & Helme, 1974). However, the influence of these ubiquitous agents on cell proliferation in human tumours and on volumetric changes in neoplasms does not appear to have been reported. Serial observations of human tumours growing as xenografts in immune-suppressed mice provide an opportunity to explore the hormonal factors controlling the progression of cancer, without the constraints necessarily surrounding human experimentation. This paper reports some preliminary observations on the influence of biogenic amines and amine-receptor-blocking drugs on the growth of human colorectal carcinomas in xenografts.

### MATERIALS AND METHODS

*Xenograft technique.*—Female CBA/lac mice were immunosuppressed by the technique of Steel *et al.* (1978). This technique involves thymectomy followed 2 weeks later by injection of cytosine arabinoside (Cytostar, the Upjohn Company) at a dose of 200 mg/kg and, after a further 24 h, the administration of 9 Gy of whole-body irradiation from a $^{60}Co$ source. Pre-treatment with cytosine arabinoside obviates the need for marrow reconstitution after irradiation. Small fragments (2–3 mm in greatest linear dimension) of tumours HXK4 and HXK7 (Nowak *et al.*, 1978) were implanted s.c. in each flank of the mice. Tumour HXK4 was originally propagated from a moderately well differentiated carcinoma of the rectosigmoid junction, and tumour HXK7 was originally propagated from a moderate to poorly differentiated carcinoma of the rectum.

*Tumour measurement.*—Starting on the 20th day after implantation, tumours were measured every 1–2 days. The largest and

* Present address: Department of Anatomy, Monash University, Clayton 3168, Victoria, Australia.

smallest superficial diameters were recorded, and the tumour volume was calculated as (mean diameter)$^3\pi/6$. The daily volume of each tumour ($V_t$) was divided by the volume of that tumour on the first day of measurement ($V_0$) to obtain the relative tumour volume. The mean and s.e. of this quotient were then plotted as a function of time after the first measurement of each control and experimental group of tumours. The relative volume was calculated because inter-tumour variation in this parameter arises only during the period of measurement. The statistical significance of apparent differences between the relative volume of various groups of xenografts at a particular time after the start of treatment was assessed using the Mann–Whitney, non-parametric test for ranked observations (Sokal & Rohlf, 1969).

The control group for Tumour HXK4 consisted of 40 xenografts and the control group for Tumour HXK7 consisted of 10, each group being measured for 12 days. The amines and amine-receptor antagonists administered to experimental groups of mice are listed in the Table. Each experimental group consisted of 5–6 mice bearing 9–12 xenografts. All drugs were given by i.p. injection, and all treatments began on the 20th day after implantation.

### RESULTS

### *The influence of adrenergic agonists and antagonists*

The administration of adrenaline, a broad-spectrum $\alpha$-, $\beta_1$- and $\beta_2$-adrenergic agonist, produced short-term suppression of the growth of Xenograft Line HXK4, mean tumour volume reaching a nadir on the second day of treatment (Fig. 1). Conversely, treatment with sotalol, a $\beta_1$- and $\beta_2$-adrenergic antagonist, accelerated tumour growth (Fig. 3). The phosphodiesterase inhibitor, theophylline, prolonged the adrenaline-induced inhibition

TABLE.—*Biogenic amines and related drugs tested for influence of xenograft growth (all agents given by i.p. injection from the 20th day after implantation)*

| Agent | | | | | |
|---|---|---|---|---|---|
| Approved pharmacological nomenclature | Chemical nomenclature | Pharmacological actions | Dose (mg/kg) | Schedule (No. of doses/ day) | Tumour tested |
| Adrenaline | 3,4-dihydroxy-$\alpha$[(methylamino) methyl]benzyl alcohol | $\alpha$, $\beta_1$ and $\beta_2$ adrenergic agonist | 1·0 | 3 | HXK 4 |
| Terbutaline | 1-(3,5-dihydroxyphenyl)-2-tert-butylamine ethanol sulphate | $\beta_2$ adrenergic agonist | 10 | 3 | HXK 4 |
| Sotalol | N-[4-[1-hydroxy-2[(1-methyl-ethyl)amino]ethyl]phenyl] methane sulphonamide | $\beta_1$ and $\beta_2$ adrenergic antagonist | 20 | 2 | HXK 4 |
| Practolol | 4-(2-hydroxy-3-isopropyl-aminoproxy) acetanilide | $\beta_1$ adrenergic antagonist, adrenergic agonist, local anaesthetic | 20 | 3 | HXK 4 |
| Theophylline | 1,3-dimethylxanthine ethylenediamine | Phosphodiesterase inhibition, increase in membrane permeability to $Ca^{++}$ | 150 | 3 | HXK 4 |
| Serotonin | 5-hydroxytryptamine creatinine sulphate | Serotoninergic agonist | 0·1<br>0·01 | 3<br>3 | HXK 4<br>HXK 4 |
| BW 501C | $\alpha$-anilino-N-2-chlorphenoxy-propyl chlorophenoxypropyl-acetamidine hydrochloride monohydrate | Serotoninergic antagonist | 5 | 3 | HXK 4 |
| Dimiprit | S-[3-(N,N-dimethylamino) propyl]isothiourea | Histamine $H_2$-receptor agonist. Diamine oxidase inhibitor | 1·0 | 3 | HXK 4 |
| Cimetidine | N″-cyano-N-methyl-N′-[2-(5-methylimadaxol-4-yl) methylthioethyl]guanidine | Histamine $H_2$-receptor antagonist | 5 | 3 | HXK 4<br>HXK 7 |

FIG. 1.—Graph of relative tumour volume *vs* time after start of treatment. Tumour Line HXK4. Control ......, Adrenaline ▪-▪-▪, Adrenaline and theophylline •—•—•—•, Terbutaline — — — —. * Indicates a statistically significant difference ($P < 0·05$) between the volume of control and experimental xenografts.

FIG. 3.—Graph of relative tumour volume *vs* time after start of treatment. Tumour Line HXK4. Control ......, Sotalol ▬▬▬, Practolol ------. * Indicates a statistically significant difference ($P < 0·05$) between control and experimental xenografts.

FIG. 2.—Graph of relative tumour volume *vs* time after start of treatment. Tumour Line HXK4. Control ······, Theophylline — — —. No statistically significant difference between the control and treated xenografts.

of tumour growth (Fig. 1) but theophylline in the absence of xenogenous adrenaline appears to have had little effect on tumour growth (Fig. 2).

Administration of terbutaline, a selective $\beta_2$-adrenergic antagonist, produced little if any inhibition of tumour growth (Fig. 3) whereas practolol, a selective $\beta_1$-adrenergic antagonist, promoted tumour growth to a similar extent to sotalol (Fig. 3).

*The influence of serotonergic agonist and a serotoninergic antagonist*

Treatment of mice with serotonin, at a dose of either 0·01 or 0·1 mg/kg, failed to influence the rate of growth of Xenograft HXK4 (Fig. 4). By contrast, the anti-serotoninergic drug BW 501C had a small inhibitory effect on Tumour HXK4 (Fig. 5) and a greater and more prolonged inhibitory effect on Tumour HXK7 (Fig. 6).

FIG. 4.—Graph of relative tumour volume *vs* time after start of treatment. Tumour Line HXK4. Control ......, Serotonin (0·1 mg/kg) ▬▬▬, Serotonin (0·01 mg/kg) — — —. No statistically significant differences.

FIG. 5.—Graph of relative tumour volume *vs* time after start of treatment. Tumour Line HXK4. Control ......, BW 501C — — —, Cimetidine ▬▬▬. * Indicates a statistically significant difference ($P < 0{\cdot}05$) between control and experimental xenograft.

*The influence of a histamine $H_2$-receptor agonist and a histamine $H_2$-receptor antagonist*

The histamine $H_2$-receptor antagonist Dimiprit appeared not to influence the growth of Tumour HXK4 (Fig. 7). However, the histamine $H_2$-receptor antagonist cimetidine strongly inhibited the growth of Tumour HXK4 (Fig. 5) and had a slightly inhibitory effect on Tumour HXK7 (Fig. 6).

### DISCUSSION

These results clearly suggest that the growth of human bowel cancer in xenograft can be influenced by biogenic amines, although many details of this influence remain to be elucidated. However, even at this stage, 3 issues seem to justify further discussion. These are: first, the failure of serotonin and Dimiprit to accelerate tumour growth though their antagonists inhibit tumour growth; secondly, the possible role of cyclic nucleotides in the regulation of tumour growth; and, thirdly, the mechanism of tachyphylaxis to the growth-inhibitory agents used.

Failure of treatment with serotonin or Dimiprit to accelerate tumour growth may be a feature of the doses used. In earlier experiments on the influence of serotonin on cell proliferation it was found that the effect of this agent was highly dose-dependent, low doses promoting cell division whilst higher doses were ineffective or inhibitory (Tutton, 1974; Tutton & Barkla, 1978*a*). In the case of histaminic agonists, specific desensitization of receptors may rapidly follow exposure of cells to high levels of stimulants (Barsoum & Gaddum, 1935). Alternatively, tumour growth may already be maximally stimulated by endogenous histamine or serotonin.

Cyclic nucleotides have now been implicated in the control of proliferation in a

FIG. 6.—Graph of relative tumour volume *vs* time after start of treatment. Tumour Line HXK7. Control ......, BW 501C — — —, Cimetidine ▬▬▬. * Indicates a statistically significant difference ($P<0{\cdot}05$) between control and experimental xenograft.

FIG. 7.—Graph of relative tumour volume *vs* time after start of treatment. Tumour Line HXK4. Control ......, Dimiprit •—•—•—. No statistically significant difference exists.

vast array of cell types. High intracellular levels of cyclic guanosine monophosphate (cGMP) or treatment with agents that promote the formation of cGMP are associated with rapid cell division in bacteria (Benlohr *et al.*, 1974), meristematic plant cells and mammalian fibroblasts (Goldberg *et al.*, 1974), haemopoietic stem cells (Byron, 1974), lymphocytes (Hadden *et al.*, 1970), granulocyte-macrophage progenitor cells (Kurland *et al.*, 1977), epidermal cells (Voorhees *et al.*, 1973) and intestinal epithelial cells (Tutton, 1976). Cyclic adenosine monophosphate (cAMP), on the other hand, has been shown to inhibit division in many cell types (for a review see Whitfield *et al.*, 1976) and is an important mediator in the process of contact inhibition of cell division (Pastan *et al.*, 1973). The results in the present communication are compatible with the hypothesis that tumour growth is promoted by intracellular cGMP, the synthesis of which is inhibited by blockade of serotonin or histamine receptors. Conversely cAMP, the synthesis of which is activated by $\beta$-adrenergic agonists such as adrenaline, may inhibit tumour growth.

The rapid tachyphylaxis to injected amines and amine antagonists which was seen in the xenograft experiments may have a pharmacological basis or may be dependent upon selected growth of cells which are resistant to the drugs used. Xenografts may contain subpopulations of cells with differing pharmacological responses and thus, when growth of some but not all of these subpopulations is even permanently suppressed, tumour growth will be resumed because of relative expansion of the subpopulation of resistant cells. Pharmacological factors that could feasibly be responsible for the observed tachyphylaxis include changes in receptor sensitivity or change in the activity of the phosphodiesterases which are responsible for degrading cyclic nucleotides. Changes in receptor sensitivity after administration of amines has been most extensively studied with respect to the influence of $\beta$-adrenergic agonists on adenyl cyclase activity. In this system desensitization due to reduction in the number of functioning drug receptors (Mukherjee *et al.*, 1975; Mickey *et al.*, 1975), reduction in receptor affinity (Lin *et al.*, 1977) and negative receptor–receptor cooperation (Limbird *et al.*, 1975) have been demonstrated. In addition to membrane receptor changes, tachyphylaxis may be mediated by a specific cAMP-phosphodiesterase, the synthesis of which is induced by high intracellular levels of cAMP (Appleman & Terasaki, 1975). The latter explanation does appear to be relevant, at least for the observed tachyphylaxis to adrenaline, since a phosphodiesterase inhibitor prolonged the suppressive effect of this amine.

We gratefully acknowledge the support and encouragement of Professor M. J. Peckham and the technical assistance of Mr Ted Merryweather and Mr John Gibbs.

### REFERENCES

Appleman, M. M. & Terasaki, W. L. (1975) Regulation of cyclic nucleotide phosphodiesterase. *Adv. Cyclic Nuc. Res.*, **5**, 153.

Barsoum, G. C. & Gaddum, J. H. (1935) The pharmacological estimation of adenosine and histamine in blood. *J. Physiol.*, **85**, 1.

Benlohr, R. W., Haddox, M. E. & Goldberg, N. D. (1974) Cyclic 3′5′ guanosine monophosphate in *Escherichia coli* and *Bacillus licheniformis*. *J. Biol. Chem.*, **249**, 4329.

Bullough, W. S. & Laurence, E. B. (1966) Accelerating and decelerating actions of adrenalin on epidermal mitotic activity. *Nature*, **210**, 715.

Byron, J. W. (1972) Evidence for a $\beta$-adrenergic receptor initiating DNA synthesis in haemopoietic stem cells. *Exp. Cell Res.*, **71**, 228.

Byron, J. W. (1975) Manipulation of the cell cycle of the hemopoietic stem cell. *Exp. Haematol.*, **3**, 44.

Byron, J. W. (1977) Mechanism for histamine $H_2$-receptor induced cell-cycle changes in the bone marrow stem cell. *Agents Actions*, **7**, 209.

Epifanova, O. I. & Tchoumak, M. G. (1963) On the action of adrenaline on the mitotic cycle of intestinal epithelium in mice. *Tsitolgii*, **5**, 455.

Goldberg, N. D., Haddox, M. K., Dunham, E., Lopez, C. & Hadden, J. W. (1974) The yin yang hypothesis of biological control: Opposing influences of cyclic GMP and cyclic AMP in the regulation of cell proliferation and other biological processes. In *The Cold Spring Harbor Symposium on the Regulation of Proliferation in Animal Cells*. Eds Clarkson & Baserga. New York: Cold Spring Harbor Laboratories. p. 40.

Hadden, J. W., Hadden, E. M. & Middleton, E. (1970) Lymphocyte blast transformation. I. Demonstration of adrenergic receptors in human peripheral lymphocytes. *Cell. Immunol.*, **1**, 583.

Hunt, H. & Tutton, P. J. M. (1976) Adrenergic factors influencing the mitotic rate in stratified squamous epithelium of the buccal mucosa of the rat. *Clin. Exp. Pharmacol. Physiol.*, **3**, 207.

Klein, R. M. (1977) Alteration of cellular proliferation in the ileal epithelium of suckling and weaned rats: the effect of isoproterenol. *Cell Tiss. Kinet.*, **10**, 353.

Kurland, J. I., Hadden, J. W. & Moore, M. A. S. (1977) Role of cyclic nucleotides in the proliferation of committed granulocyte-macrophage progenitor cells. *Cancer Res.*, **37**, 4534.

Leeson, S. J. & Voaden, M. J. (1970) A chalone in the mammalian lens. II. Relative effects of adrenaline and noradrenaline on cell division in the rabbit lens. *Exp. Eye Res.*, **9**, 67.

Limbird, L. E., De Meyts, P. & Lefkowitz, R. J. (1975) $\beta$-adrenergic receptors: evidence for negative cooperativity. *Biochem. Biophys. Res. Commun.*, **64**, 1160.

Lin, C. S., Hurwitz, L., Jenne, J. & Avner, B. P. (1977) Mechanism of isoproterenol-induced desensitization of tracheal smooth muscle. *J. Pharmacol. Exp. Therap.*, **203**, 12.

Mickey, J., Tate, R. & Lefkowitz, R. J. (1975) Subsensitivity of adenylate cyclase and decreased $\beta$-adrenergic receptor binding after chronic exposure to (-)-isoproterenol *in vitro*. *J. Biol. Chem.*, **250**, 5727.

Mukherjee, C., Caron, M. G. & Lefkowitz, R. J. (1975) Catecholamine-induced subsensitivity of adenylate cyclase associated with loss of $\beta$-adrenergic binding sites. *Proc. Natl Acad. Sci. U.S.A.*, **72**, 1945.

Norrby, K. (1973) Effect of heparin, histamine and serotonin on density-dependent inhibition of cell proliferation in two fibroblastic cell lines. *Virchows Archiv.* (*Cell Pathol.*), **15**, 75.

Nowak, K., Peckham, M. J. & Steel, G. G. (1978) Variations in response of xenografts of colo-rectal carcinoma to chemotherapy. *Br. J. Cancer*, **37**, 576.

Pastan, I., Willingham, M., Carchman, R. & Anderson, W. B. (1973) Cyclic AMP metabolism in normal and transformed fibroblasts. In *The Role of Cyclic Nucleotides in Carcinogenesis*. Ed. Schultz & Gratyner. New York: Academic Press. p. 47.

Sokal, R. R. & Rohlf, F. J. (1969) *Biometry*. San Francisco: W. H. Freeman & Co.

Steel, G. G., Courtenay, V. D. & Rostom, A. Y. (1978) Improved immune-suppression techniques for xenografting of human tumours. *Br. J. Cancer*, **37**, 224.

Tutton, P. J. M. (1974) The influence of serotonin on crypt cell proliferation in the jejunum of rat. *Virchows Archiv.* (*Cell Pathol.*), **16**, 79.

Tutton, P. J. M. (1976) The influence of histamine on epithelial cell proliferation in the jejunum of the rat. *Clin. Exp. Pharmacol. Physiol.*, **3**, 369.

Tutton, P. J. M. & Barkla, D. H. (1976) A comparison of cell proliferation in normal and neoplastic intestinal epithelia following either biogenic amine depletion of monoamine oxidase inhibition. *Virchows Archiv.* (*Cell Pathol.*), **21**, 169.

Tutton, P. J. M. & Barkla, D. H. (1977) The influence of adrenoreceptor activity of cell proliferation in colonic crypt epithelium and in colonic adenocarcinomata. *Virchows Archiv.* (*Cell Pathol.*), **24**, 139.

TUTTON, P. J. M. & BARKLA, D. H. (1978*a*) The influence of serotonin on the mitotic rate in the colonic crypt epithelium and in colonic adenocarcinoma in rats. *Clin. Exp. Pharmacol. Physiol.*, **5**, 91.

TUTTON, P. J. M. & BARKLA, D. H. (1978*b*) Stimulation of cell proliferation by histamine $H_2$-receptors in dimethylhydrazine-induced adenocarcinomata. *Cell Biol. Int. Rep.*, **2**, 199.

TUTTON, P. J. M. & HELME, R. D. (1974) The influence of adrenoreceptor activity on crypt cell proliferation in the jejunum of rat. *Cell Tissue Kinet.*, **7**, 125.

VOORHEES, J. J., KELSEY, W., STAWISKI, M. & 4 others (1973) Increased cyclic GMP and decreased cyclic AMP levels in rapidly proliferating epithelium of psoriasis. In *The Role of Cyclic Nucleotides in Carcinogenesis*. Ed. Schultz & Grayzner. New York: Academic Press. p. 325.

WHITFIELD, J. F., MACMANUS, J. P., RIXON, R. H., BOYTON, A. L., YOUDALE, T. & SWIERENGA S., (1976) The positive control of cell proliferation by the interplay of calcium ions and cyclic nucleotides. A review. *In vitro*, **12**, 1.